# Maternal Overnutrition Programs Central Inflammation and Addiction-Like Behavior in Offspring

**DOI:** 10.1155/2018/8061389

**Published:** 2018-06-20

**Authors:** Larisa Montalvo-Martínez, Roger Maldonado-Ruiz, Marcela Cárdenas-Tueme, Diana Reséndez-Pérez, Alberto Camacho

**Affiliations:** ^1^Department of Biochemistry, College of Medicine, Universidad Autónoma de Nuevo Leon, San Nicolás de los Garza, NL, Mexico; ^2^Department of Cell Biology and Genetics, College of Biological Sciences, Universidad Autónoma de Nuevo Leon, San Nicolás de los Garza, NL, Mexico; ^3^Neurometabolism Unit, Center for Research and Development in Health Sciences, Universidad Autónoma de Nuevo Leon, San Nicolás de los Garza, NL, Mexico

## Abstract

Obesity or maternal overnutrition during pregnancy and lactation might have long-term consequences in offspring health. Fetal programming is characterized by adaptive responses to specific environmental conditions during early life stages. Programming alters gene expression through epigenetic modifications leading to a transgenerational effect of behavioral phenotypes in the offspring. Maternal intake of hypercaloric diets during fetal development programs aberrant behaviors resembling addiction in offspring. Programming by hypercaloric surplus sets a gene expression pattern modulating axonal pruning, synaptic signaling, and synaptic plasticity in selective regions of the reward system. Likewise, fetal programming can promote an inflammatory phenotype in peripheral and central sites through different cell types such as microglia and T and B cells, which contribute to disrupted energy sensing and behavioral pathways. The molecular mechanism that regulates the central and peripheral immune cross-talk during fetal programming and its relevance on offspring's addictive behavior susceptibility is still unclear. Here, we review the most relevant scientific reports about the impact of hypercaloric nutritional fetal programming on central and peripheral inflammation and its effects on addictive behavior of the offspring.

## 1. Introduction

According to the World Health Organization, nearly 39% of adults aged 18 years and over were overweight in 2016, and 13% were obese. Maternal obesity adversely impacts both maternal and offspring health, increasing the susceptibility to show metabolic abnormalities later in life such as obesity, dyslipidemia, type 2 diabetes mellitus, and hypertension as well as behavioral disorders related to schizophrenia, autism, and compulsive eating disorders [1, 2]. Maternal obesity or maternal overnutrition programs metabolic and hormonal nodes that modulate neuronal development during embryogenesis. For instance, neuronal maturation, including axonal pruning, synaptic plasticity, and stable tract formation between structures, is selectively programmed during pregnancy and lactation by consumption of high-sugar, high-fat, or high-sugar-high-fat diet formulas. Under this scenario, nutritional programming defines after-weaning selective behavioral phenotypes in offspring that might be exacerbated during adulthood, such as incentive-motivation behaviors leading to compulsive eating disorders.

Maternal obesity or overnutrition during fetal programming activates molecular and cellular mechanisms that command a new physiological state that might compromise basic metabolic and neuronal homeostasis. Metabolic imbalance during obesity can lead to overactivation of the immune system, triggering a process of chronic inflammation evidenced in animal models and humans. In fact, nutritional programming by hypercaloric diets promotes an inflammatory phenotype that contributes to disrupted energy sensing pathways in metabolic-relevant systems including adipose tissue, liver, pancreas, muscle, and the brain. Inflammation in the brain is associated with a cross-talk between peripheral and central cell types that potentially activates microglia in the brain. However, it is still unclear which immune cells infiltrate into the fetal brain leading to microglia activation and neuroinflammation during maternal nutritional programming. Moreover, it is unknown if maternal overnutrition during fetal programming originates central inflammation by microglial activation in the absence of peripheral immune cells infiltration. Finally, the role of neuroinflammation during maternal nutritional programming and its effects on defective behavior related to compulsive eating disorders in the offspring have only started to be dissected. In this review, we will discuss the role of maternal programming on peripheral and central immune cross-talk and its relevance in the development of incentive-motivation behavior such as addiction in the offspring.

Obesity is a metabolic condition showing positive energy balance driven by several factors including human genetics, life style, environment, body activity, and diet [6]. At first, obesity was conceived as a metabolic disorder showing an increase in white adipose tissue mass-modulated exclusively by the peripheral nervous system. However, recent evidence shows that the central nervous system (CNS) plays a major role in the modulation of adipose tissue mass and function. Also, the CNS is a major regulator of food intake and metabolism and seeks for rewards such as food; in a pathological state, CNS activation might lead to addiction-like behavior [7].

## 2. Obesity Is a Potential Deregulator of Energy-Satiety Integration in the CNS, Leading to Overfeeding 

In nature, all living organisms require energy to sustain life and perform activities. Energy is mainly provided by food assigned into three main formulas such as proteins, carbohydrates, and fats. However, energy surplus such as overnutrition disrupts metabolic and hormonal homeostasis and has harmful consequences in health. On this matter, metabolic and hormonal signals from the periphery arrive to the CNS to give a message about the energy balance of the body. The CNS integrates these signals through evolutionary-conserved neuronal tracts connecting peripheral organs to selective brain structures. For instance, the brainstem receives information from the gut, while the hypothalamus integrates circulating/humoral signals. The brainstem and hypothalamus control satiety and integrate these signals by saying when and how much to eat [7]. However, under a pathological scenario satiety might be overestimated, leading to activation of the reward system, which modulates the incentive motivation to work and search for food despite an “apparent” satiety signal. The reward system integrates dopaminergic neurons located in the ventral tegmental area (VTA) that project to the nucleus accumbens (NAc) and also innervate several regions of the prefrontal cortex (PFC), central amygdala, basolateral amygdala (BLA), and hippocampus and dopamine neurons in the substantia nigra (SN) that project to the dorsal striatum, which has traditionally been associated with appetitive learning, performance, and motivation [8]. It has been hypothesized that incentive motivation for palatable food contributes to overfeeding during obesity [9, 10]. For instance, an evasion of satiety has been identified in obesity leading to positive energy balance and an increase of body mass index [11, 12]. Several researchers propose that, like drug-addiction, obesity sets an altered motivational behavior for seeking foods rich in fat and sugars (hypercaloric) that have high reward value (pleasant), which is potentially dependent on dopamine neurotransmission [10].

The proposal that overeating during obesity might be considered as food addiction in the Diagnostic and Statistical Manual of Mental Disorders (DSM) was initially based on phenotypic similarities between patterns of overeating in obese individuals and drug abuse in addicted persons. It has been shown in both animal models and humans that repeated exposure to high-fat or high-sugar diets disrupts the integration of energy-satiety peripheral signals into the CNS, leading to overfeeding which indeed shares behavioral similarities to addiction [10, 13]. For instance, obese humans might experience the following: (1) enhanced motivation over hypercaloric food intake including recurrent and excessive consumption, (2) an increased time spent seeking palatable food in contrast to habitual activities, and (3) frequent or permanent relapse to hypercaloric foods after dieting. Of note, obesity or palatable food overconsumption might be subject-specific and it is potentially modulated by environment-subject interaction. Based on this proposal, researchers have applied a selective Yale Food Addiction Scale (YFAS) to provide a standardized measure for the assessment and diagnosis of food addiction based mainly on substance dependence criteria [14, 15]. These evidences suggest that overfeeding during obesity might be related to incentive motivation for palatable food in human and animals. However, are humans born addicted or do they become addicted to food?

## 3. Potential Role of Fetal Programming by Hypercaloric Food in the Development of Addictive Behaviors in the Offspring

Exposure to hypercaloric foods impacts the individual metabolic-hormonal settings of mothers and fathers and might also affect their offspring. Substantial scientific evidence has demonstrated the detrimental role of hypercaloric food intake during pregnancy and lactation leading to a failure in metabolic homeostasis, favoring the development of incentive-motivation behavior for food in the offspring. In this context, epidemiological data from human catastrophes such as the Dutch famine (1944), the siege of Leningrad (1942–1944), the great Chinese famine (1958–1961), and also the Överkalix study (1890-present) have shown that changes in diet intake regarding overfeeding or fasting are associated with disorders in the offspring such as diabetes mellitus type 2 and cardiovascular diseases [16–19]. In addition, there is evidence of behavioral alterations, including schizophrenia [20–22], affective disorders [23], and addiction [24, 25]. Based on this data, Barker (1998) proposed the “Barker hypothesis” suggesting that the transgenerational effect of diet exposure during pregnancy modulates metabolic and behavioral phenotypes in the offspring, a mechanism known as “fetal programming” [26]. In specific, this hypothesis claims that oversupply or absence of energy intake during embryonic-fetal development provides the fetus with physiological adaptations to a new milieu of metabolic-hormonal threshold to face a potential adverse postnatal environment similar to those whose parents were exposed to fetal programming. In fact, any stimulus or insult throughout embryonic-fetal development, including stress, infections, substance abuse, overnutrition, and behavioral alterations, might result in molecular adaptations that produce permanent structural, physiological, and metabolic changes in the fetus. Also, fetal programming might increase the risk of serious physiological problems in perinatal stages including miscarriage, fetal-congenital anomalies, thromboembolism, and gestational diabetes.

**Figure 1:**
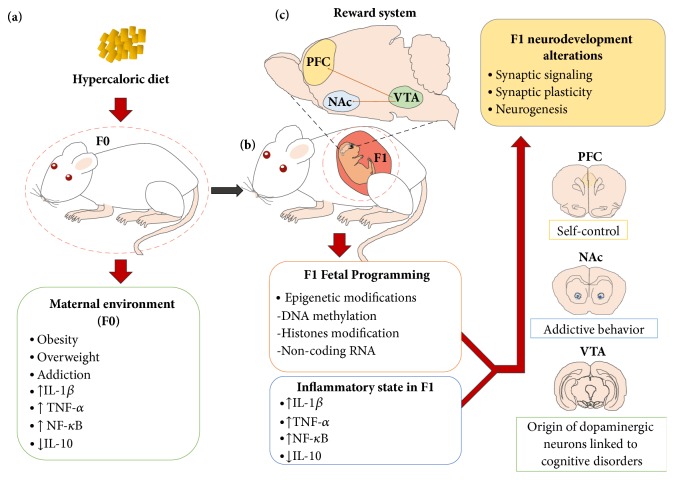
Maternal programming by hypercaloric diets increases the development of addictive behavior in offspring. (a) Hypercaloric diet intake or obesity during pregnancy leads to activation of an inflammatory state of mothers (F0), favoring aversive intrauterine environment and selective increase in proinflammatory markers. (b) Positive inflammatory state in mothers correlates with the one in the offspring (F1) and also with activation of fetal epigenetic program including DNA methylation, histone modification (ip acetylation, methylation, sumoylation, and ubiquitination), and noncoding RNAs (ip miRNA, piRNA, and lncRNA), which are capable of altering gene expression profile in selective regions belonging to the reward system: prefrontal cortex (PFC), nucleus accumbens (NAc), and ventral tegmental area (VTA). (c) Epigenetic programming associates with neurodevelopment alterations in F1 including synaptic signaling, synaptic plasticity, and neurogenesis which contributes to addictive-like behavior.

Initial reports demonstrated that energy-dense food disrupts the appetite-energy sensing and satiety systems, exacerbating the reward for food. Also, excessive consumption of palatable food can lead to a profound hyposensitivity to reward, leading to compulsive eating behavior similarly observed during drug seeking [27]. Nutritional programming in murine models induces alterations in behavior and synaptic plasticity, favoring higher consumption and sensitization to alcohol, methamphetamines, and cocaine [28, 29]. Maternal exposure to hypercaloric diets during pregnancy or lactation has shown to increase the long-term preference for junk food in the offspring [30], potentially associated with repeated, intermittent increases in extracellular dopamine (DA) in the NAc and the VTA [31–35]. Molecularly, these synaptic plasticity changes show greater expression of the ΔFosB gene [28] and both dopamine (e.g., DR2, DAT) and opioid pathway genes expression (e.g., the *μ*-opioid receptor) at early stages of development [35–39]. In fact, the opioid pathway regulates the rewarding effects of palatable food; an injection of *μ*-opioid agonists in the NAc increases preference for high-fat or sugar-rich foods [40, 41], and its antagonists decrease palatable food predilection, even at doses that show no effect on standard food intake [42, 43]. In addition, fetal programming by drugs such as nicotine exposure during pregnancy might also disrupt brain gene expression involved in neuronal glutamatergic (e.g., GluA1, GluA2, and CaMKII*α*) and dopaminergic (e.g., DR2, DAT, and DR1) signaling plasticity in the hippocampus [44, 45], the laterodorsal tegmental nucleus [46], and the NAc [47–49]. In murine maternal overnutrition models, a transcription modulation of glutamatergic and dopaminergic systems in the NAc and prefrontal cortex that increases fat/sugar food preferences in the offspring has been identified [50–53] (Figure 1).

In humans, it is still under investigation if obesity or maternal overnutrition during pregnancy leads to the development of food addiction in the offspring. Initial reports found that children from obese mothers or mothers with eating disorders presented more binge eating and night eating [54, 55] and consumed more carbohydrates compared to their normal weight counterparts [56]. Also, a positive correlation between addicted parents and food addiction in their offspring has been proven [57], although epidemiological studies are needed to support these findings.

## 4. Obesity and Maternal Overfeeding Promote Peripheral and Central Inflammatory Response 

An organism's first line of defense is the innate immune system and it includes physical barriers such as the skin, specific cell types such as macrophages, and complement proteins. The second line of defense is the inflammatory response which consists of an innate cellular system and humoral response that happens during injury to restore physiological homeostasis. The function of the immune system is not limited to fighting infections and repairing tissue damage, it also plays a key role in shaping neuronal tracts during CNS development. For instance, cells from the innate immune system, such as microglia, regulate neurogenesis, synaptic plasticity, and synaptic striping, and are the major antigen-presenting cells (APC) in the CNS [58]. Cross-talk between peripheral immune system and brain-resident immune cells is in part regulated by B and T lymphocytes, macrophages, and antibodies which migrate and penetrate the blood-brain barrier (BBB) [58, 59] and have also been reported in the cerebrospinal fluid [60]. Under an altered physiological scenario, peripheral immune cells located in the brain become proinflammatory entities and secrete cytokines promoting an exacerbated immune response by microglia. By doing this, peripheral immune cells integrate positive feedback with microglia that modulates neural growth and development [61]. Microglial cells are the brain-resident macrophages of the CNS; these oversee surveillance of the CNS integrity and respond to pathogens and injuries and also to very subtle alterations in their microenvironment [62]. In healthy brains, microglia remain in a ramified stated, and when activated, they enlarge their cell body, change to a phagocytic state, and execute similar functions to those of other tissue-resident macrophages such as proinflammatory cytokines secretion, antigen presentation, and ROS production and phagocytosis, leading to neuroinflammation [63]. Under this scenario, neuroinflammation is beneficial because it removes debris or dysfunctional neurons; under a pathological state, however, microglia activation is upregulated and can be damaging to neurons because of the excessive release of ROS and proinflammatory cytokines, leading to neuroinflammation [64], and might contribute to tissue damage and disease pathology. Although microglial activation could be harmful, microglia are necessary to provide essential trophic factors for the survival of neurons, like brain-derived neurotrophic factor (BDNF) and glial-derived neurotrophic factor (GDNF) [63].

An atypical form of inflammation, primarily induced by fatty acids accumulation in selective energy-dependent tissues including liver, adipose tissue, muscle, and brain occurs when there is a positive energy balance, as found in obesity or maternal overnutrition, [65]. Fatty acids promote a type of body inflammation termed “metainflammation” or “metabolic inflammation,” which involves several immune cells (T, B lymphocytes and microglia) and proinflammatory mediators (cytokines and chemokines) leading to metabolic and neurodegenerative disorders [66, 67]. Obesity or overnutrition during pregnancy leads to maternal immune activation (MIA), which favors changes in plasma and placental tissue-specific lipidomic profile, recruiting lipid species to membrane Toll-Like Receptor 4 (TLR4) and activating the nuclear factor-kappa B (NF-*κ*B) pathway, through an increase of TLR2, TLR4, IL-6, IL-18, and TNF-*α* mRNA levels and macrophage markers cluster of differentiation including (CD)11b, CD14, and CD68 [68–70]. Maternal overnutrition in a sheep model of obesity demonstrated that inflammatory markers such as CD-68, TGF-*β*1, and TNF-*α* found in the mother are also identified in the offspring after birth [71]. These evidences show that imbalances in the peripheral and immune system cross-talk might compromise early stages of CNS development and differentiation potentially contributes to epilepsy, schizophrenia, cerebral palsy, Parkinson disease (PD), Alzheimer disease (AD), and ASD [72].

## 5. Central-Peripheral Immune System Cross-Talk Regulates Synaptic Plasticity, Neurogenesis, and Neurodegeneration

**Figure 2:**
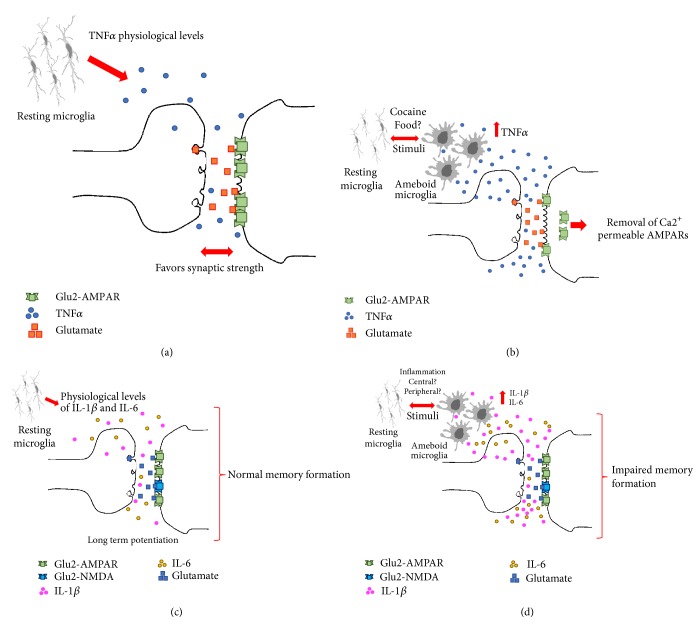
Synaptic modulation by cytokine levels. (a) According to Bettie and cols. in 2002 and Stellwagen and cols. in 2006 results TNF-*α* was demonstrated to promote normal expression of AMPARs favoring synaptic strength at excitatory synapses. (b) In contrast, high levels of TNF-*α* can affect synaptic plasticity by removing of Ca2+-permeable AMPARs from plasma membrane according to Lewitus et al., 2014 (b). (c) Physiological levels of IL-1*β* and IL-6 are detrimental to maintain and modulate hippocampus activity [3, 4]. (d) Excess of IL-1*β* and IL-6 affect LTP and impair memory formation [3, 5].

As mentioned before, neuroinflammation is how the innate immune system protects the CNS and controls initial stimulus, and although it is intended to be protective and beneficial, out-of-control inflammation can be fatal and contribute to tissue damage and disease pathology, including neurodegeneration [73]. Substantial scientific evidence has shown that neuroinflammation commands changes in cerebral plasticity and influences synaptic function and memory [72]. However, there are no conclusive data showing a cause-effect relationship in terms of the role of cytokines on synaptic plasticity modulation in the NAc, as a trigger to addictive behavior. Some data from neuroimmune interactions in humans in the behavioral response to drugs have shown small clues about their clinical relevance; however, these studies do not identify the molecular mechanisms of such interactions [74, 75]. Cause-effect relationship is even harder to identify because the CNS shows a selective cytokine profile when compared to peripheral cytokines during drug exposure. For instance, Calipari and colleagues did not find differences between the TNF-*α* expression in serum when mice were exposed to cocaine [76]. However, Lewitus et al. identified TNF-*α* release from microglia after cocaine consumption as a potential regulation of neuronal and behavioral plasticity during both the induction and expression of drug-induced behaviors [77]. Molecularly, initial reports have shown that glia releases TNF-*α* to fit surface expression of AMPARs, favoring the synaptic strength at excitatory synapses [78, 79]. In fact, TNF-*α* stimulation in brain slices reduced corticostriatal synaptic strength by removing of Ca2+ permeable AMPARs from the plasma membrane [80], suggesting that high levels of TNF-*α* affect synaptic plasticity, but physiological levels favor brain development and synaptic strength [79] (Figure 2). These evidences reinforce the previous hypothesis that TNF-*α* profile in the CNS modulates synaptic plasticity during addiction; however, it is still unknown whether central TNF-*α* accumulation might be a node of inflammatory activation during addiction or there is a cause-effect relationship involving a cross-talk between peripheral and central mechanism.

Synaptic plasticity during addiction might also be modulated by chemokines. For instance, the CCL4 and CCL17 chemokines and their ligand CCL25 are upregulated in microglia following morphine treatment and might also induce local chemotaxis of axon terminals or dendritic spines. Blocking the synthesis of certain chemokines prevents the relapse of drug-seeking behavior following drug reexposure months later [81]. In addition, the modulation of the G-CSF chemokine concentration in the NAc and the prefrontal cortex is crucial to alter the motivation of cocaine consumption but not of food [76]. Together, chemokines have a very profound neuromodulatory effect on the regulation of neural circuits involved in addiction, but the molecular mechanisms on this scenario still need to be described in detail.

Central and peripheral immune cross-talk response might also modulate hippocampal neurogenesis. It has been reported that when CD4^+^ T lymphocytes are systemically depleted in mice, there are reduced hippocampal neurogenesis, learning impairment, and a decrease in BDNF secretion in the brain, whereas inoculation of CD4^+^ T lymphocytes promotes hippocampal neurogenesis and restores the deficits observed before [82, 83]. In models of autoimmune encephalitis in which inflammatory responses are upregulated, synaptic plasticity in the hippocampus and long-term potentiation (LTP) are affected because of the high levels of IL-1*β*. While peripheral inflammatory responses related to TNF-*α* and IL-6 have been identified to modulate hippocampus activity [84, 85], it has not been fully described whether central IL-1*β* comes from infiltrating lymphocytes or activated microglia within the CNS [86]. In any case, central physiological levels of IL-1*β* are detrimental, because excessive cytokines level or blockage of IL- 1 signaling through IL-1ra has been reported to cause memory impairment [87], potentially due to Ca^2+^ influx throughout NMDA receptors, Src kinases activation, and NR2A/B subunits phosphorylation [88].

As mentioned before for TNF-*α* and IL-1*β*, initial reports characterized that the IL-6 cytokine is required in normal physiological levels for the formation of LTP, but levels above normal affect memory formation and learning [89, 90]. Supporting those findings, IL-6 KO mice display memory impairment [84] and show reduced proliferating neuronal stem cells (NSCs) in the hippocampus [91]. Under physiological conditions, IL-6 is related to neural plasticity and NSCs proliferation through the JAK/STAT, MAPK/cAMP, Ras-MAPK, and PI3K pathways [92]. Lastly, the immune system is involved in regulation of hippocampal neurogenesis, through the kynurenine pathway [93], affecting directly the hippocampal neurogenesis and memory and learning processes [82] (Figure 2).

A positive inflammatory profile including the IL-1, IL-6, IL-1*β*, TNF-*α*, and IFN-*γ* has been evidenced in a wide range of neurodegenerative diseases. In particular, TNF-*α* production by active microglia is highly related to IFN-*γ* secreted by T cells into the CNS parenchyma [3]. Secreted IFN-*γ* and other inflammation mediators activate the MEK/ERK signaling pathway leading to TNF-*α* production and neuronal death [4]. Also, TNF-*α* and IFN-*γ* lead to microglial nitric oxide (NO) production, favoring oxidative stress, inhibiting neuronal respiration, and favoring neuronal apoptosis [94]. Of note, these responses seem to be independent, although there might be a node at different time-frame. For instance, the TNF-*α* targets its own receptor to activate the NF-*κ*B-dependent proinflammatory pathway and the expression of inducible NO synthase (iNOS) that mediates NO production, which is regulated by NF-*κ*B and STAT transcription factors [95].

In addition, IL-6 also might potentially favor neuronal death. By promoting the formation of the IL-6/IL-6R/gp130 complex, the proinflammatory IL-6 activates the JAK1, JAK2, and TYK2 pathway and promotes the phosphorylation of STAT1 and STAT3. In specific, IL-6 favors neuronal death by the STAT1 nuclear transcription factor, which associates with transcription of IFNs, including type I (IFN-*α*, IFN-*β*, and IFN-*ω*) and type II (IFN-*γ*), all of them involved in detrimental innate immune responses. However, IL-6 might also be anti-inflammatory and promote cell survival through STAT3-PI3K/Akt signaling activation by regulating cell cycle proteins (CDK2), antiapoptotic proteins (Bcl-2), and proapoptotic proteins (Bax, Bad, and caspase-9) [5, 96]. Thus, IL-6 secretion by activated microglia regulates inflammatory mediators and shows a dual role that prevents or promotes neuronal death.

In summary, because of their dual roles associated with threshold levels, cytokines in the brain may help to maintain cerebral homeostasis having a beneficial effect in normal physiological conditions; uncontrolled or chronic neuroinflammation, however, leads to neurodegeneration, disruption of cerebral plasticity, cognitive changes, and the appearance of aberrant behavior, also defined as “sickness behavior” by Cibelli and colleagues [74].

## 6. Epigenetic Mechanisms during Fetal Programming and Transgenerational Inheritance of Addictive Phenotype

By now, we have shown evidences that metabolic inflammation during pregnancy leads to chronic inflammation in offspring that might disrupt synaptic plasticity and set a self-perpetuating cycle of addictive excessive consumption of natural and synthetic rewards [28, 97]. Despite the lack of epidemiological studies addressing the impact of human obesity or maternal overnutrition as a node to promote addiction-like phenotypes in offspring, recent evidence suggests that addiction in humans might have a common root from transgenerational inheritance, given that biomarkers that share identity in drug programming and addictive behavior have been previously described [98–101]. A transgenerational inheritance of addiction, eating disorders, or pathological phenotypes in offspring is modulated directly by epigenetics, which programs and adapts organisms in the early stages of development to face sudden changes in the environment, compared with evolutionary and natural selection processes, which tend to be slow [102]. Epigenetics refer to the mechanisms of long-term or stable regulation of gene expression that do not involve a change in gene sequences including DNA methylation and histone modification such as acetylation, sumoylation, ubiquitination, and methylation, as well as gene expression regulated by noncoding RNAs, miRNAs, piRNAs, and lncRNA [103]. The role of maternal programing by overnutrition during pregnancy and its effects on epigenetics linked to a proinflammatory profile in offspring have not been deeply analyzed yet. Initial work has shown that epigenetics modulate the immune system and potentially affects the CNS during pregnancy [103]. A recent paper identified that fat diet primes an epigenomic reprogramming of myeloid progenitor cells that led to an increased proliferation and enhanced innate immune responses [104], which the authors named as ‘‘innate immune memory” or ‘‘trained immunity,” able to mediate metabolic reprogramming for prolonged periods of time [105]. On this context, using a fetal-programing murine model with hypercaloric diet, Edlow et al. (2016) identified a significant number of deregulated miRNAs implicated in immune and proinflammatory processes, death cellular, adipogenesis and cellular stress in the males of offspring [106] (Figure 1). In humans, a positive correlation between body mass index (BMI) during prepregnancy with adiposity and inflammatory markers in offspring at birth has been demonstrated [107]. Also, Alexander and col. (2018) reported alterations in the methylation profile of genes involved in mitochondrial function, DNA repair, oxidative stress and inflammation in the placenta of children who were born from mothers with type 2 diabetes mellitus [108]. Of importance, a study in humans revealed that an adherence to Mediterranean diet for 5 years ended up in a significant increase in DNA methylation which positively correlated with a reduction in the concentrations of TNF-*α*, sICAM-1, and CRP [109].

These evidences suggest that gestational exposure to high-fat and high-sugar diets in murine models results in a disruption of the central reward system, which correlates with epigenetic biomarkers found in addicted-programed subjects. Programming sets the brain to potentiate changes in neuroplasticity [97, 110], neuronal death, neurotoxicity [111], etc., in postnatal life, which leads to the development of cognitive disorders such as addiction [112–115]. It is still under investigation if humans share the aberrant reward plasticity that has been described in murine models.

## 7. Conclusions

Maternal overnutrition leads to fetal adaptive responses which sets a neuronal gene expression program in offspring. Selective intake of diets rich in fats and sugars at critical stages recruit central and peripheral inflammatory cell type markers, such as microglia and T and B cells, which disrupt energy sensing and behavioral pathways increasing the susceptibility to show aberrant behaviors similar to addiction. In fact, an addictive phenotype might be transgenerational when inherited by selective epigenetic activation during fetal programming. These evidences propose the immune system activation by nutritional programming during gestation as a node to modulate changes in neuroplasticity related to incentive behavior phenotypes which must be addressed deeply for a better understanding.
